# Autoimmune Heparin-Induced Thrombocytopenia

**DOI:** 10.3390/jcm12216921

**Published:** 2023-11-03

**Authors:** Theodore E. Warkentin

**Affiliations:** 1 Department of Pathology and Molecular Medicine and Department of Medicine, Michael G. DeGroote School of Medicine, McMaster University, Hamilton, ON L8N 3Z5, Canada; twarken@mcmaster.ca; Tel.: +1-(905)-527-0271 (ext. 46139); 2 Service of Benign Hematology, Hamilton Health Sciences (General Site), Hamilton, ON L8L 2X2, Canada; 3 Transfusion Medicine, Hamilton Regional Laboratory Medicine Program, Hamilton, ON L8L 2X2, Canada

**Keywords:** autoimmune heparin-induced thrombocytopenia, disseminated intravascular coagulation, heparin-independent platelet-activating antibodies, platelet factor 4, thrombosis

## Abstract

Autoimmune thrombocytopenia (aHIT) is a severe subtype of heparin-induced thrombocytopenia (HIT) with atypical clinical features caused by highly pathological IgG antibodies (“aHIT antibodies”) that activate platelets even in the absence of heparin. The clinical features of aHIT include: the onset or worsening of thrombocytopenia despite stopping heparin (“delayed-onset HIT”), thrombocytopenia persistence despite stopping heparin (“persisting” or “refractory HIT”), or triggered by small amounts of heparin (heparin “flush” HIT), most cases of fondaparinux-induced HIT, and patients with unusually severe HIT (e.g., multi-site or microvascular thrombosis, overt disseminated intravascular coagulation [DIC]). Special treatment approaches are required. For example, unlike classic HIT, heparin cessation does not result in de-escalation of antibody-induced hemostasis activation, and thus high-dose intravenous immunoglobulin (IVIG) may be indicated to interrupt aHIT-induced platelet activation; therapeutic plasma exchange may be required if high-dose IVIG is ineffective. Also, aHIT patients are at risk for treatment failure with (activated partial thromboplastin time [APTT]-adjusted) direct thrombin inhibitor (DTI) therapy (argatroban, bivalirudin), either because of APTT confounding (where aHIT-associated DIC and resulting APTT prolongation lead to systematic underdosing/interruption of DTI therapy) or because DTI inhibits thrombin-induced protein C activation. Most HIT laboratories do not test for aHIT antibodies, contributing to aHIT under-recognition.

## 1. Introduction

**Figure 1:**
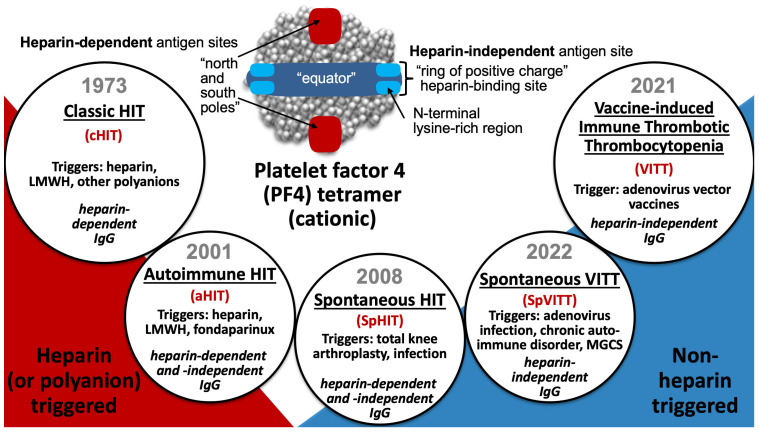
Five platelet-activating anti-PF4 disorders. Red text indicates abbreviations.

This review discusses a subcategory of the adverse drug reaction, heparin-induced thrombocytopenia (HIT), known as “autoimmune HIT”, or aHIT. I define aHIT as an entity in which proximate exposure to heparin (unfractionated heparin [UFH], low-molecular-weight heparin [LMWH], or fondaparinux) is implicated, and where clinical/laboratory and/or serological features indicate that heparin-independent, platelet-activating properties are present. As the laboratory with which I am associated—the McMaster Platelet Immunology Laboratory—performs the serotonin-release assay (SRA) as its main platelet activation test, I will sometimes use the term “heparin-independent serotonin-release” (HISR) when referring to this special property of aHIT antibodies. Based on recent breakthroughs in our understanding of how anti-PF4 disorders can be triggered and the different types of anti-PF4 antibodies, aHIT can be considered one of five recognized anti-PF4 disorders (Figure 1) [1,2]. The term “autoimmune HIT” was introduced into the literature 12 years ago [3].

Whereas classic HIT (cHIT) and aHIT are triggered by heparin, and vaccine-induced immune thrombotic thrombocytopenia (VITT) is triggered by adenovirus vector vaccines, “spontaneous” HIT (SpHIT) and “spontaneous” VITT (SpVITT) refer to aHIT- and VITT-mimicking disorders, respectively, that are not triggered by heparin or vaccination. In this respect, laboratory tools to distinguish between HIT and VITT antibodies are required to distinguish between SpHIT and SpVITT.

The above definition of aHIT, which requires proximate exposure to heparin, differs somewhat from certain other previously used definitions, for example, where some authors (including myself) have included SpHIT as an aHIT disorder [4,5,6]. However, SpHIT is an anti-PF4 disorder with HIT-mimicking clinical and serological features that—by definition—occurs in the absence of proximate exposure to heparin or another polyanionic pharmaceutical agent [7]. Further, it is now recognized that there are HIT-mimicking disorders in which the pathogenic platelet-activating anti-PF4 antibodies more closely resemble those seen in VITT [8,9,10]; for example, VITT-like antibodies have been detected in some patients with monoclonal gammopathy of clinical significance [11,12], as well as following symptomatic adenovirus infection [13,14] or unspecified viral infection (SpVITT) [15]. Thus, in this review, I will only consider a patient as having aHIT if there is established (or strongly suspected) proximate exposure to UFH (or LMWH or fondaparinux) that is believed to be responsible for the generation of the pathogenic antibodies and the ensuing prothombotic, thrombocytopenic disorder.

Table 1 lists five HIT scenarios associated with aHIT antibodies. Thrombosis frequency appears to be unusually high in aHIT. For example, the overall frequency of HIT-associated thrombosis is approximately 40% to 70% [16,17,18]. However, for aHIT, the frequency of thrombosis is likely much higher, at least 75%, and perhaps >90% (discussed subsequently). Although this could reflect recognition bias (thrombosis drawing attention to aHIT diagnosis), there is a paucity of reported patients with a clear aHIT diagnosis who did not develop clinically-evident thrombosis. Further, whereas the median platelet count nadir in HIT is approximately 50 to 60 × 10^9^/L [17,19], in aHIT the median platelet count nadir is approximately 20 × 10^9^/L, and there is a high frequency of associated overt disseminated intravascular coagulation (DIC) (discussed subsequently).

## 2. Methods

**Figure 2:**
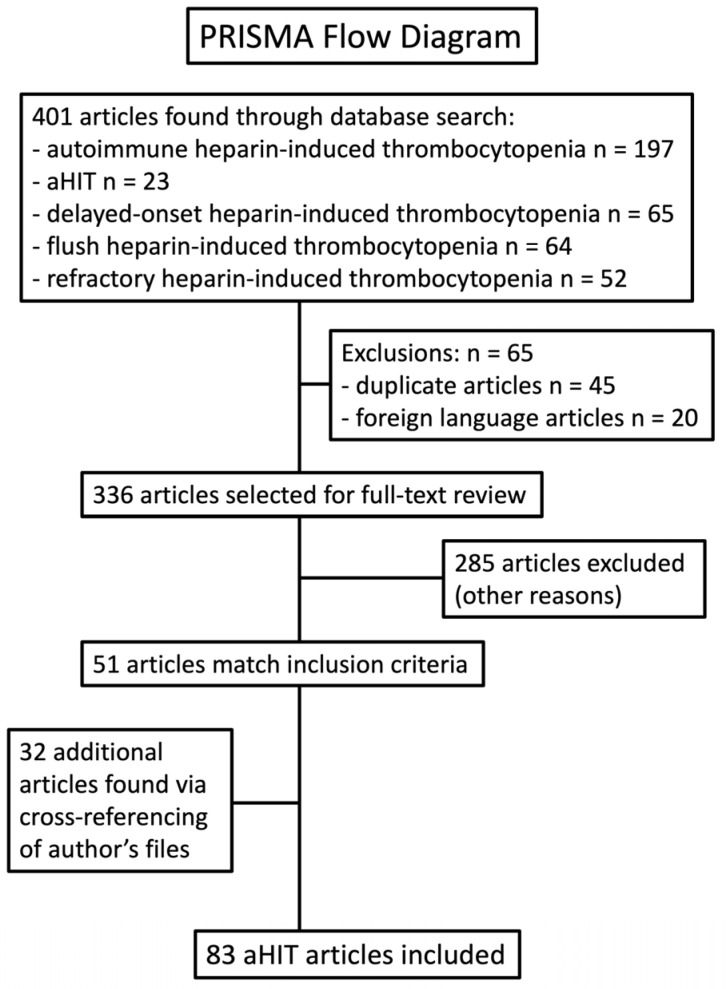
PRISMA flow diagram to identify reports describing patients with aHIT.

A systematic review was performed using PRISMA guidelines (Figure 2). The five search terms were “autoimmune heparin-induced thrombocytopenia”, “aHIT”, “delayed-onset heparin-induced thrombocytopenia”, “flush heparin-induced thrombocytopenia”, and “refractory heparin-induced thrombocytopenia”. As discussed later in this review, I chose the latter three terms because these entities are recognized as being aHIT disorders. Except for one Japanese publication [20] of historical note, we only included articles written in the English language. Papers were included if—in the judgment of the author—they described one or more cases that appeared to meet clinical criteria for one of the five aHIT disorders listed in Table 1; in addition, cases were identified in which laboratory data was available supporting the presence of HISR (for laboratories such as McMaster Platelet Immunology that perform the SRA) or (for other laboratories) other indicators of heparin-independent platelet activation.

Cases were reviewed, and some cases reported as aHIT were rejected based upon judgment of a more compelling diagnosis. For example, a patient labeled as aHIT had a clinical course suggesting an alternative diagnosis of SpHIT beginning approximately one week following shoulder arthroplasty (without anticoagulant thromboprophylaxis) [21]. Another report of aHIT was confounded by treatment by cyclophosphamide for vasculitis (compelling alternative explanation for prolonged thrombocytopenia) [22]. Another case report provided insufficient documentation to determine whether aHIT was present [23]. Some cases were excluded as no testing for HIT antibodies was performed, and the diagnosis of HIT was deemed uncertain [24].

## 3. Results

I will discuss, in sequence, the five recognized aHIT disorders, laboratory diagnosis, pathogenesis, and treatment considerations.

### 3.1. Five aHIT Disorders

Among the five entities listed in Table 1, it is logical that the first three listed—“delayed-onset HIT”, “persisting (refractory) HIT”, and heparin “flush” HIT—are aHIT disorders, since they cannot plausibly have wholly heparin-dependent thrombocytopenia based on the absence (or trivial doses) of heparin. Similarly, it seems logical that fondaparinux-associated HIT would be an aHIT disorder, given that HIT antibodies in general do not cross-react with fondaparinux (discussed subsequently). Whether unusually severe HIT represents aHIT is less clear. In the following sections, I discuss these five aHIT disorders in more detail. The key unifying theme is that all aHIT disorders feature HISR resulting from unusually pathogenic anti-PF4 antibodies with heparin-independent platelet-activating properties (heparin-dependent antibodies are usually also identifiable).

### 3.2. Delayed-Onset HIT

#### 3.2.1. Terminology

The emergence of HIT as an immune-mediated adverse effect of heparin that featured thrombocytopenia and thrombosis dates to 1973 [25], with the terms “heparin-induced thrombocytopenia” and “heparin-associated thrombocytopenia” used most often in the earliest reports [25,26,27,28,29,30,31,32,33,34]. In general, these studies emphasized the heparin-dependent nature of this disorder, with rapid platelet count recovery upon heparin discontinuation.

However, beginning in the 1980s, the term “delayed-onset HIT” was used in two papers from two different Australian groups [35,36]. For them, “delayed-onset HIT” had a meaning that differs from the current use of this term. The seminal paper by Beng Chong and colleagues [35] distinguished between HIT of “delayed onset”—occurring after 8 or more days of exposure to heparin, and with thrombotic complications in five of the six patients reported, and in which he identified platelet-activating antibodies of IgG class—and a different patient group characterized by early-onset, generally mild and transient thrombocytopenia of no clinical consequence. Although Chong initially designated the former group as “type 1” and the latter group as “type 2”, he later reversed this, namely the early transient form of thrombocytopenia as “type 1”, and the delayed (immune-mediated) group as “type 2” [37]. The aim was to distinguish clearly between early, transient, clinically non-consequential, type 1 HIT (sometimes also called “non-immune heparin-associated thrombocytopenia” [38]) with the potentially life- and limb-threatening “type 2”, or “delayed-onset”, HIT disorder mediated by platelet-activating antibodies.

The second Australian paper that also used the term “delayed-onset” in relation to immune-mediated HIT was by Van der Weyden et al. [36]; writing in the *Medical Journal of Australia*, their report was entitled “Delayed-onset heparin-induced thrombocytopenia. A potentially malignant syndrome”. They described a dozen patients in whom thrombotic events (venous, *n* = 5; arterial, *n* = 1; both venous and arterial, *n* = 1) occurred between 7 and 14 days following start of heparin therapy in seven of the 12 patients, with five other patients recognized with thrombocytopenia alone that occurred in a similar time frame. The median platelet count nadir was 52 × 10^9^/L (range, 8 to 88). Platelet aggregation studies demonstrated a heparin-dependent, platelet-activating factor in patient plasma. Their report emphasized the usual rapid correction of thrombocytopenia following heparin cessation (within a week in all patients), reinforcing the heparin-dependent nature of this adverse drug reaction. Some other papers that appeared during the 1980s also referred to immune-mediated HIT as “delayed-onset HIT” [39,40].

However, this term “delayed-onset HIT” now has two differing meanings in the history of HIT. As per the aforementioned Australian (and some other) papers, the term has been used as a general name for HIT as an immune-mediated reaction, as there is always a minimum period of time—generally at least five days—between the initial administration of the immunizing heparin exposure (arbitrarily designated as “day 0”) and the first evidence of an HIT-related platelet count fall.

The second—and now current—use of the term, “delayed-onset HIT”, dates from a 2001 paper written by myself with Professor John Kelton [41], in which we described an atypical clinical presentation of HIT in which there was a minimum five-day delay between the discontinuation of heparin and the onset of the HIT-related platelet count fall. This entity is now regarded as a form of aHIT, and is discussed in more detail in the next section.

#### 3.2.2. Delayed-Onset HIT as an aHIT Disorder

**Figure 3:**
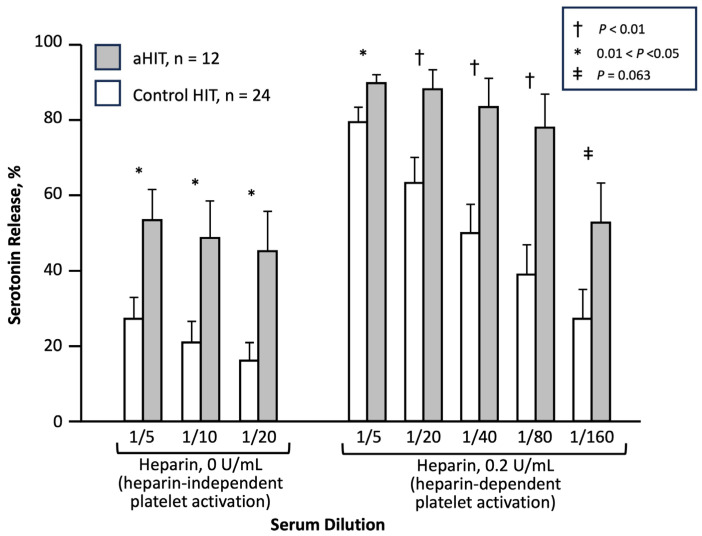
aHIT sera show significantly greater heparin-independent serotonin-release (HISR) versus control HIT sera. Modified from [41], with permission (copyright, 2001, The American College of Physicians, Philadelphia, PA, USA).

I consider 2001 as the year that the first aHIT disorder—“delayed-onset HIT”—was first established, as a Brief Communication published in the *Annals of Internal Medicine*, entitled, “Delayed-onset heparin-induced thrombocytopenia and thrombosis” [41]. This article is highlighted in a historical context for several reasons. First, this was not a single case report, but rather a series of 12 patients with an atypical presentation of HIT, six presenting as in-patients and six presenting as outpatients. Second, the case definition required that there be a minimum of 5 days between the last heparin exposure, and the first evidence of a platelet count decline related to HIT; in some cases, the first evidence of HIT was a thrombotic event occurring after discharge from hospital, at which time unexpected thrombocytopenia was newly identified. Since heparin has a relatively short half-life (approximately 60 min [42]), a 5-day gap between the last exposure to this drug, and the beginning of an event (platelet count fall, thrombosis) means that no heparin would be remaining in the patient, arguing strongly for a drug-independent platelet-activating effect. And third, this study also compared the SRA profiles between the 12 patients with delayed-onset HIT and 24 control subjects; the study found that heparin-independent platelet-activating properties, i.e., HISR at 0 U/mL heparin, was significantly greater in the patients with delayed-onset HIT (Figure 3). This observation provided a pathophysiological rationale for the concept of aHIT, pointing to the existence of aHIT antibodies (discussed subsequently).

Another reason 2001 is an appropriate year for recognition of the aHIT disorder, delayed-onset HIT, is that there was concomitant recognition of such an atypical presentation of HIT by investigators in the United States, led by Lawrence (Larry) Rice, MD [43]. He and his collaborators identified a series of 14 patients who presented late after their last heparin exposure, including patients whose platelet count fell after stopping heparin. Although both the McMaster and USA papers were submitted simultaneously to the same journal, the publication of the USA paper was delayed until 2002 [43]. Although the Rice paper included two patients who presented with late thrombosis without thrombocytopenia (cases which would not have met the case definition in the Warkentin and Kelton 2001 paper), it is possible that such patients might have had an unrecognized period of thrombocytopenia following hospital discharge, with platelet count recovery, prior to the onset of thrombosis, and then an abrupt platelet count drop with heparin resumption; indeed, exactly such a clinical profile has been reported [44].

Within five years, two further events solidified the concept of “delayed-onset HIT” presenting after heparin cessation. In January 2006, Jackson and colleagues [45] published four such cases in the journal, *Vascular and Endovascular Surgery*, noting that “[t]hese reports have been [previously] confined to the internal medicine literature”, thus broadening the reach to include the surgical community. Also, in December 2006, the US Food and Drug Administration (FDA) notified health-care professionals of revisions to the WARNINGS section of the prescribing information for heparin, to inform clinicians of the possibility of the delayed onset of HIT [46,47]. Thus, by 2006, the concept of delayed-onset HIT became more widely accepted.

Viewing these three publications together [41,43,45], with 30 patients reported, the following features are evident. First, patients typically presented with thrombosis, rather than initial recognition of unexpected thrombocytopenia (all 30 patients had at least one HIT-associated thrombotic event). Second, the magnitude of thrombocytopenia was variable, with some patients presenting with severe thrombocytopenia, but others with mild thrombocytopenia; indeed, this latter group was especially likely to receive further therapeutic-dose heparin administration (to treat the thrombotic event), inevitably prompting abrupt declines in the platelet count, a phenomenon known as “rapid-onset HIT” [48,49]. Third, the spectrum of thrombosis was remarkably wide, ranging from venous (predominantly, deep venous thrombosis [DVT] and pulmonary embolism [PE]), but also uncommon venous thromboses such as renal and adrenal vein thrombosis (the latter manifesting as adrenal hemorrhage), to arterial thrombosis (strokes, myocardial infarction, and limb artery thrombosis), with some patients having both venous and arterial thrombosis. Fourth, some patients had overt DIC, with hypofibrinogenemia and microthrombosis. Of note, one patient [43] had been exposed to heparin solely through heparin flushes, an entity discussed in more detail later (see Section 3.4, Heparin Flush HIT).

**Figure 4:**
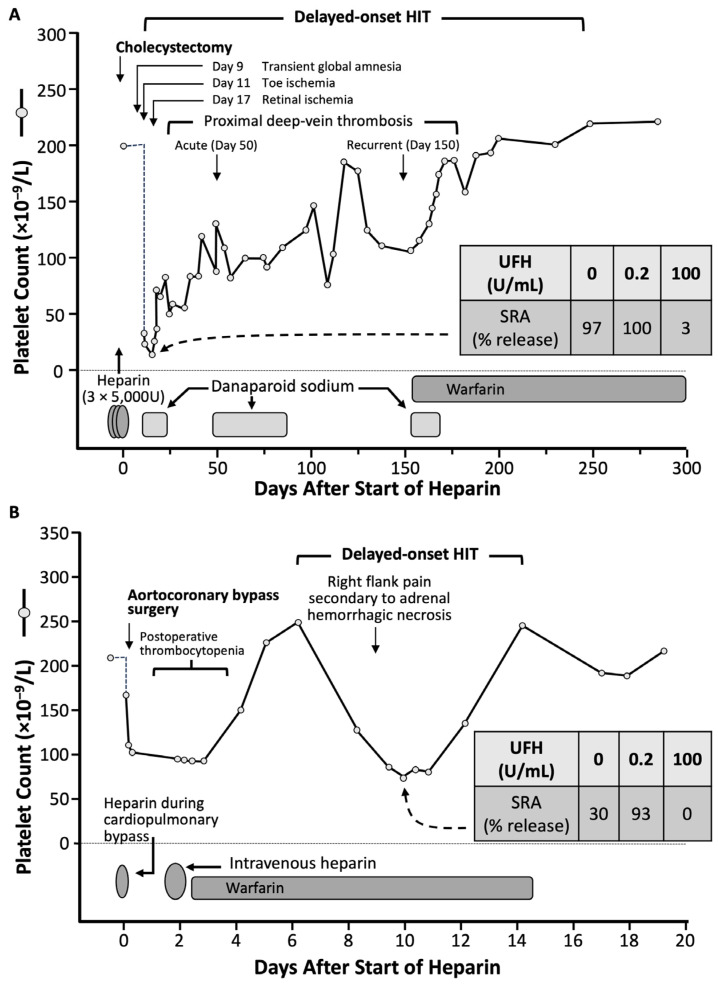
Two patients with aHIT. (**A**,**B**) The inset shows results of the serotonin-release assay (SRA), with per cent (%) serotonin-release at different concentrations of unfractionated heparin (UFH), for a day 10 sample. Modified from [50], with permission. (Copyright, 2002, Current Science Inc., Bengaluru, India).

Figure 4 summarizes two of the 12 patients included in the 2001 study on delayed-onset HIT [41], as presented in more detail subsequently [50]. The first patient developed ischemic events starting one week after receiving three postoperative injections of UFH post-cholecystectomy. She developed protracted thrombocytopenia and episodes of recurrent thrombosis lasting approximately 250 days. The second patient developed marked but transient thrombocytopenia one week post-cardiac surgery, and abdominal pain; imaging showed an adrenal hemorrhage.

The term “delayed-onset HIT” is a misnomer, as the timing of onset of this form of aHIT is likely the same as seen in classic HIT (cHIT) [19,51]. Figure 4 shows that both patients’ platelet count falls began during the day 5 to 10 “window” characteristic of HIT [44,48]. Thus, the key point is that the presence of aHIT (heparin-independent) antibodies, and the ensuing platelet count fall that occurs, worsens, or persists, when heparin is not being administered, is the key to understanding this atypical disorder.

#### 3.2.3. Definition of Delayed-Onset HIT

Although one of the inclusion criteria for delayed-onset HIT in the *Annals of Internal Medicine* [41] paper was a minimum 5-day interval between the last heparin exposure and the onset of thrombocytopenia or thrombosis, this was a definition of convenience with an aim to help establish a rational clinical basis for why highly pathological, heparin-independent aHIT antibodies could be present. Logically, however, such aHIT antibodies could be present irrespective of any given temporal relationship between heparin exposure and associated platelet declines. Accordingly, the definition of delayed-onset HIT was subsequently broadened to include patients whose platelet count decline either began, or that worsened, despite stopping heparin [4,52,53]. In addition, the concept of “refractory” or “persisting” HIT—in which platelet counts do not quickly recover after heparin cessation—also became part of the recognized aHIT spectrum. Indeed, many patients manifest both atypical aspects of HIT: for example, Figure 4A shows clearly that the same patient whose platelet count fell several days after stopping heparin (delayed-onset HIT) also had thrombocytopenia that persisted for many months (persisting or refractory HIT).

#### 3.2.4. Delayed-Onset HIT Reports with aHIT Antibodies

In this section, I list and review several studies, including case series and case reports, describing patients with delayed-onset HIT where there is laboratory evidence of heparin-independent platelet-activating properties, i.e., the presence of aHIT antibodies (Table 2) [41,54,55,56,57,58,59,60,61,62,63,64,65,66,67,68,69,70,71,72,73,74,75,76,77,78]. The majority of these papers are from the McMaster University Platelet Immunology Laboratory (Hamilton, ON, Canada), given that it has been routine—since the invention of the SRA [79,80]—to perform the SRA both in the absence and presence of heparin, often performed with four different heparin concentrations (0, 0.1, 0.3, and 100 IU/mL); hence, this table also refers to the laboratory phenomenon of HISR. The first paper listed in Table 2 [41] is the aforementioned study that showed significantly greater HISR in the 12 patients with delayed-onset HIT. (Note that the inhibition of platelet activation in the presence of very high heparin [100 U/mL] is as much a feature of aHIT as it is with cHIT as well as other anti-PF4 disorders, e.g., VITT, SpHIT.)

Table 2 also includes a case-series of patients diagnosed with HIT during a 38-month period (ending in March 2009) in a hospital in Hamilton; that paper describes the detailed SRA results, including data on HISR (>50% serotonin-release at 0 IU/mL UFH was considered evidence for aHIT antibodies) [54]. This paper noted that this phenomenon of heparin-independent platelet-activating properties was associated with delayed-onset thrombocytopenia in several patients, as well as delayed recovery of the platelet counts. It also suggested particularly severe clinical outcomes in patients with aHIT; for example, the phenomenon of “activated partial thromboplastin time (APTT) confounding” was seen in one of these patients (discussed subsequently in Section 3.9.2. Choice of Anticoagulation).

Table 2 also includes a study of patients with aHIT-complicating heparin “flush” exposure (discussed subsequently in Section 3.4) [55]; in this study, serum from all four patients with heparin flush HIT showed strong (>80%) HISR, whereas only approximately one-third of controls exhibited this phenomenon. This provides strong evidence that HISR is associated with aHIT antibodies associated with heparin flush HIT. Padmanabhan and colleagues [56] also showed that three patients with SRA-positive refractory HIT had aHIT antibodies that activated platelets in the presence of unusually low concentrations of PF4. Also, in a study [57] of 129 patients who developed postcardiac surgery HIT over a 30-year period, three patients who presented with thrombocytopenia and thrombosis post-discharge all had aHIT antibodies that exhibited HISR. Rollin et al. also found evidence for more severe HIT when HISR was >30% [58].

Table 2 also highlights four papers [55,59,60,61] showing data supporting an inverse relationship between HISR and platelet counts. The concept is that as HISR decreases—either gradually over time or abruptly following treatment with high-dose IVIG—the platelet count increases in a corresponding fashion. A parallel concept in immunohematology is the inverse relationship between autoreactive antibodies and thrombocytopenia in post-transfusion purpura (PTP) [81].

Table 2 includes data from 50 patients for whom laboratory evidence for aHIT antibodies was provided. Most of the studies utilized the McMaster Platelet Immunology Laboratory SRA, except for three using the HIPA test [69,75,77], one the PEA (with low PF4 concentrations) [56], and one a test for ATP release [78]. For the HIPA test, strong heparin-independent platelet activation was shown by platelet aggregation by 5 min with buffer control. Many of the patients listed in Table 2 had unusual complications of HIT, such as marked thrombocytopenia (platelet count nadir <20 × 10^9^/L), microvascular ischemia, venous limb gangrene, bilateral adrenal hemorrhages, and overt DIC, among others.

**Figure 5:**
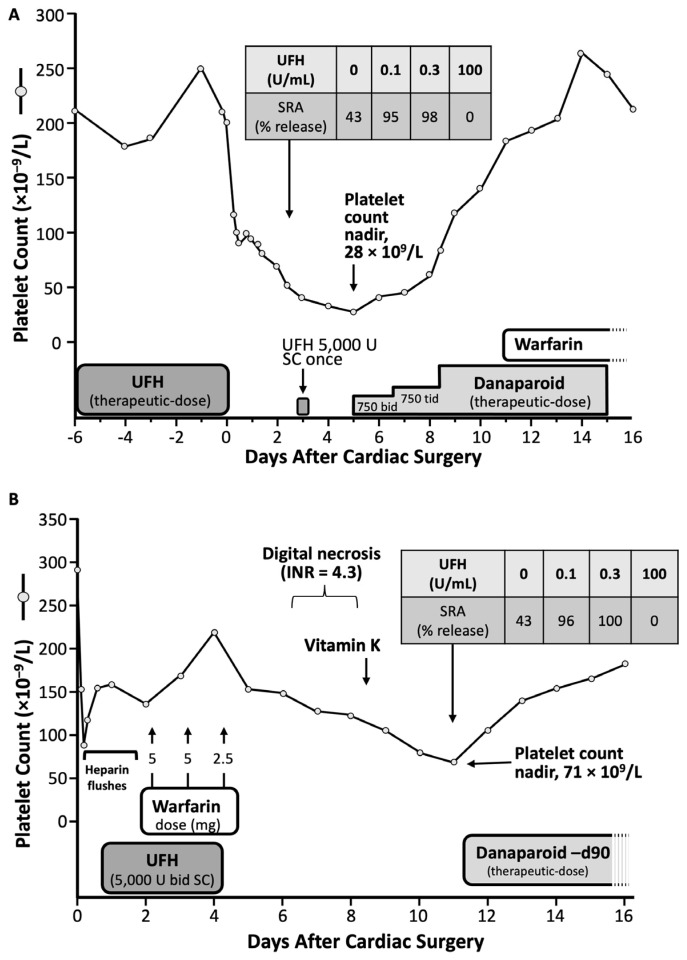
Two aHIT patients with moderate (43%) HISR. (**A**) Modified from [63], with permission (copyright 2007 International Society on Thrombosis and Hemostasis, Carborro, NC, USA). (**B**) Modified from [62], with permission (copyright 2003 Wiley-Liss, Inc., Hoboken, NJ, USA).

Two patients listed in Table 2 [62,63] are shown in Figure 5. These cases point out that the degree of HISR is not necessarily >80% (both patients had 43% HISR). This suggests that there could be *platelet-dependent* factors underlying aHIT, i.e., a patient with aHIT antibodies bearing only moderate HISR activity could still develop aHIT if their platelets are unusually reactive to aHIT antibodies. This is discussed later in Section 3.8.2, Patient (Platelet) Risk Factors for HIT.

#### 3.2.5. Delayed-Onset HIT Reports without Laboratory Documentation of aHIT Antibodies

Many papers have described patients with aHIT diagnosed on clinical grounds (atypical presentation), without laboratory demonstration of aHIT antibodies (Table 3).

Table 3 lists several notable clinical features, including unusually severe thrombocytopenia in some patients, occurrence of DIC with hypofibrinogenemia, development of warfarin-associated venous limb gangrene, and so forth. A high proportion of patients were recognized following discharge from the hospitalization in which the immunizing heparin had occurred (these are listed as “post-D/C” in the Table). Numerous patients shown failed argatroban treatment, most often based on new or progressive thrombosis while on argatroban therapy. A high proportion of the patients are listed as having delayed (“refractory”) platelet count recovery, a topic considered briefly in the next section.

### 3.3. Persisting (Refractory) HIT

The term “persisting HIT” or “refractory HIT” refers to patients whose platelet count recovery seems unduly prolonged. Since the median time to platelet count recovery—following cessation of heparin—is approximately 3 to 4 days [44] for cHIT, with approximately 90% recovering in 7 days time, one definition of refractory HIT would be a time to platelet count recovery greater than 1 week. Indeed, there are some examples in the HIT literature of much longer platelet count recoveries. For example, I reported a patient whose platelet count took 16 weeks to reach consistently above 150 × 10^9^/L, and even longer to reach the usual baseline platelet count (see Figure 3A).

### 3.4. Heparin Flush HIT

It has long been recognized that exposure to heparin in small amounts, such as through maintenance of intravascular catheters, can result in formation of heparin-dependent (cHIT) anti-PF4 antibodies. For example, Dr. Elizabeth Ling and I [112] described two patients who developed rapid-onset HIT following administration of a heparin bolus administered 9 and 12 days following orthopedic surgery; for both patients, their only known heparin exposure was UFH flushes through an intraarterial catheter used only during the surgery. In one patient, in whom daily blood samples were available, it could be shown that platelet-activating anti-PF4 antibodies became detectable on postoperative day 6. It is important to emphasize that both patients required therapeutic-dose heparin (via bolus administration) to develop clinical manifestations of HIT (post-bolus rapid-onset thrombocytopenia with acute anaphylactoid reactions). Neither of these cases, however, represented aHIT, as the antibodies were heparin-dependent.

Mayo and colleagues [113] investigated systematically whether flushes were associated with anti-PF4/heparin antibody formation; they found a high frequency of anti-PF4/heparin antibodies by enzyme-linked immunosorbent assay (ELISA), with 1/49 patients testing SRA positive; none developed overt thrombocytopenia, indicating that seroconversion was not associated with formation of aHIT antibodies. Gettings and coworkers [114] identified 19 critically-ill patients who had detectable anti-PF4/heparin antibodies, most in association with heparin flush exposures, some of whom may have had HIT; however, as they studied a critically-ill patient population, and provided only summary data, it was difficult to discern whether any of their patients had had aHIT.

There are numerous papers describing HIT associated with the sole immunizing exposure to heparin being flushes only [43,111,115,116,117,118,119,120,121,122,123,124] (patients receiving heparin flushes with marrow transplantation are discussed later). Implicated doses of heparin range from a single injection of heparin given periprocedurally (e.g., a single 1000-unit heparin flush for implantable cardioverter defibrillator [ICD] implantation [123]) to a few hundred units given daily over one or a few days [111,115,116,117,118,119,121,124] to intermittent small doses of UFH given at weekly intervals [120]. One study reported that the only heparin exposure was administered to “lock” the arterial and venous ports of the hemodialysis catheter [122]; experimental models do support the potential for “leakage” of heparin into the systemic circulation when used for locking hemodialysis ports [125].

A variety of associated venous and arterial thrombotic events were reported, most often DVT [43,55,116,117,118,119,121,123], sometimes complicated by PE [43,116]. Interestingly, when the HIT-associated thrombotic complication was an upper-extremity DVT, this was generally the result of flushing a catheter that had been placed in the ipsilateral limb [55,117,122,124]. This observation is consistent with the known strong association—in patients with proven HIT—between upper-limb DVT and recent/concurrent placement of an intravascular catheter in the same limb as developed the symptomatic DVT [126]. More unusual thrombotic events that were reported included cerebral venous sinus thrombosis (CVST) [111,121], renal vein thrombosis [116], mesenteric vein thrombosis [111,119,120], arterial stroke [118,122], and acute limb ischemia [124]. The frequency of thrombosis among these reported cases was 13/15 (87%) [43,111,115,116,117,118,119,120,121,122,123,124].

#### 3.4.1. Heparin Flushes during Stem Cell Transplantation

Tezcan et al. [127] first reported HIT following bone marrow transplantation, one autologous, one allogeneic—with heparin flushes implicated; one patient developed upper-extremity DVT. Subsequently, two studies [55,128] made the striking observation that HIT occurred in approximately 4% of patients undergoing autologous stem cell transplantation for multiple myeloma and amyloidosis (pooled data, 9/222 = 4.1%, i.e., 5/121 and 4/101]). This strikingly high frequency could reflect such factors as granulocyte-colony stimulating factor (G-CSF) administration and discontinuation of pretransplant cyclophosphamide conditioning (i.e., absence of immunosuppression that would have otherwise occurred). Five of nine (55.6%) patients developed thrombosis, most often upper-extremity DVT at the apheresis catheter site (*n* = 4), although one patient required emergency vascular surgery for limb-threatening aorto-iliac artery thrombosis. All nine patients tested SRA- and ELISA-positive, with median optical density (OD) of 2.7 OD units.

The study by Mian and colleagues [55] noted that all four patients had heparin-independent platelet-activating properties (>80% HISR), a phenomenon that was significantly more frequent than in other patients diagnosed with HIT in hospitals of the same medical community (4/4 vs. 34/100; *p* = 0.0161). These data support the concept that assessment of heparin-independent platelet-activating properties is a marker of aHIT.

In addition, three other single case reports [129,130,131] of HIT complicating heparin flush administration in the context of preparation for stem cell transplantation have been reported; these reports also noted the use of G-CSF (filgrastim), with two patients also receiving plerixafor (agent that mobilizes peripheral blood stem cells); together with the 4% frequency of HIT in the context of heparin flush exposure mentioned earlier, these observations suggest that the proinflammatory effects of these agents may dramatically increase the frequency of HIT for what otherwise would be a rare occurrence. These three patients also developed thrombotic complications: right coronary artery thrombotic occlusion resulting in STEMI [129], DVT complicated by transmetatarsal limb amputation [130], and DVT with saddle PE [131]; indirect support for heparin flush HIT being an aHIT disorder was seen in the case reported by McKenzie and colleagues [129], where prolonged thrombocytopenia eventually resulted in application of high-dose IVIG (discussed subsequently). It is notable that high-dose IVIG was not effective in the report by Bavli et al. [130], with plasma exchange being required for platelet count recovery.

Overall, nine (64.3%) of the 14 patients identified in these reports [55,127,128,129,130,131] of heparin flush HIT in the context of stem cell transplantation developed one or more thrombotic events.

Stephens and colleagues [132] did not find any benefit to heparin flushes for preventing thrombosis for central venous catheter (CVC) maintenance for patients undergoing apheresis collection of peripheral blood stem cells (of interest, these investigators did find a higher frequency of catheter-related thrombosis among patients who received G-CSF). This raises the general issue as to whether heparin flushes are helpful in preventing catheter thrombosis.

#### 3.4.2. Are Heparin Flushes Helpful in Maintaining Catheter Patency?

An interesting issue is whether heparin flushes are helpful in preventing thrombotic events, at least in some of the settings in which they are used. Mitchell and colleagues [133], performing a systematic review of heparin flushes, concluded: “[o]ur search for primary literature confirms that the evidence base on heparin flushes for maintaining patency of [central venous access devices] is small and of low quality”. In another systematic review, Zhong and colleagues found no difference in long-term catheters, but found a slight advantage to heparin (versus normal saline) flushes for short-term CVC management [134]. Kordzadeh and colleagues [135] in a comprehensive review found longer duration of catheter performance for heparin versus normal saline. Another systematic review was performed by Sharma and colleagues [136]; these authors found that “[h]eparin has little favorable effects to maintain patency of catheter than normal saline but not in secondary outcomes”. The secondary outcome they referred to was that of HIT; paradoxically, there was a trend to a lower frequency of HIT among the subjects randomized to heparin flushes versus saline. This finding was based on the single trial by Schallom and colleagues [137], where two patients were ELISA-positive in the normal saline study arm, and zero patients in the UFH study arm. The authors noted that these patients were exposed to other sources of heparin. However, it is perhaps noteworthy that we published a preliminary report that found a nonsignificant lower frequency of antibody formation in patients who received UFH (versus saline) flushes for intraoperative management [138].

### 3.5. Fondaparinux-Associated HIT

Fondaparinux is an anticoagulant modeled after the highly sulfated antithrombin-binding pentasaccharide sequence of heparin [139]. Paradoxically, this agent is both a (rare) trigger of HIT, as well as a common anticoagulant used to treat HIT [140].

#### 3.5.1. Fondaparinux and Anti-PF4 Antibodies

Given that fondaparinux is a pentasaccharide, and that minimum heparin chain lengths of approximately 12 saccharide units are required to create antigens on PF4 [141,142], it was believed that fondaparinux should not support antigens recognized by HIT antibodies. Indeed, Savi and collaborators [143] showed that HIT sera were significantly less reactive in functional platelet activation assays compared with heparin. Indeed, this difference is clinically relevant, as clinical trial experience supports a lower risk of triggering acute HIT (versus UFH and LMWH) in patients with unrecognized HIT antibodies who are treated with fondaparinux [144].

Given its small size, it was also expected that fondaparinux would not be immunogenic in clinical use. However, unexpectedly, fondaparinux was shown to have a low, but similar, rate of anti-PF4/heparin antibody formation as seen with LMWH (enoxaparin). Interestingly, anti-PF4/heparin antibodies identified in this study did not react (in a fluid-phase ELISA) against PF4/fondparinux complexes, even when the antibodies had been formed in patients who had received fondaparinux thromboprophylaxis [145]. Platelet-activating anti-PF4 antibodies generated in a post-cardiac surgery population also did not show evidence of in vitro cross-reactivity with fondaparinux [146]. Pouplard et al. [147] also found evidence of anti-PF4/heparin antibody formation in patients treated with fondaparinux. Indeed, Greinacher and colleagues [148] later provided multiple lines of evidence that fondaparinux interacts with PF4. Subsequently, Chen et al. [149] showed that at optimal concentrations PF4 can form complexes with PF4 that can be recognized by the HIT-mimicking monoclonal antibody, KKO. Nonetheless, the approximately similar frequencies of seroconversion with fondaparinux and LMWH could be a coincidence, given that stoichiometric modeling suggests that LMWH dosing is usually too high—whereas fondaparinux dosing is usually too low—to produce the optimal levels of PF4/polyanion needed to trigger an anti-PF4/polyanion immune response [150].

#### 3.5.2. Fondaparinux as a Treatment of HIT

As might be expected, given the lack of in vitro cross-reactivity of most HIT antibodies for fondaparinux, this agent ought to be an effective treatment for HIT. Initial experience using fondaparinux for HIT 20 years ago was promising [151,152,153]. This initial experience was supported by several case-series that used HIT serology to support the underlying diagnoses of true HIT, with high frequencies (>90%) of success [154,155,156,157,158]. These findings were corroborated by a systematic review [159].

However, occasional failure of fondaparinux has been reported, which in some cases appears to be related to the interaction of fondaparinux with the HIT antibodies. For example, I have reported three patients [160,161,162] who evinced both clinical and laboratory evidence of cross-reactivity with fondaparinux. One [160] patient developed HIT with associated venous thrombosis after neurosurgery (glioblastoma resection); however, thrombocytopenia persisted for approximately one week after switching from UFH thromboprophylaxis to therapeutic-dose fondaparinux. Platelet count resolution occurred after IVIG was given and fondaparinux was switched to rivaroxaban. Notably, The SRA showed both heparin-dependent and fondaparinux-dependent serotonin-release, without any evidence for aHIT antibodies. In contrast, another patient [161] with fondaparinux failure illustrated a completely different issue: that patient had a clinical picture of aHIT following UFH thromboprophylaxis; however, when fondaparinux treatment was given, the platelet count remained low and overt DIC persisted. Laboratory studies showed an aHIT profile; however, with serum dilution, fondaparinux-dependent serotonin-release exceeded that seen at buffer control [161]. This raises the issue that aHIT might be a risk factor for fondaparinux failure. Note that both of these published cases occurred in Canadian hospitals outside of Hamilton, Ontario, suggesting that clinical evidence of in vivo cross-reactivity with fondaparinux is likely uncommon (<3%), given that many dozens of patients have received fondaparinux in the author’s medical community (Hamilton), with only one example (to our knowledge) of a patient exhibiting clinical and laboratory evidence of fondaparinux cross-reactivity (without aHIT features) [162].

Pistulli and colleagues [163] reported a patient who developed HIT with UFH and LMWH in whom the platelet count continued to decline after switching to therapeutic-dose fondaparinux; these authors found laboratory evidence of increased platelet activation in the presence of fondaparinux. Sartori and Cosmi [164] also reported a case of aHIT following a single dose of UFH that was also associated with clinical evidence of fondaparinux failure (persisting thrombocytopenia, new venous thrombosis), although in vitro studies to document cross-reactivity were not performed.

#### 3.5.3. Fondaparinux-Associated aHIT

There are eight reported patients for whom fondaparinux was implicated as a plausible trigger of the HIT syndrome [165,166,167,168,169,170,171]. Six of the eight patients developed one or more thrombotic events, ranging from: bilateral adrenal necrosis (*n* = 2) [165,169], deep-vein thrombosis (*n* = 5) [165,167,168,169,171], superficial vein thrombosis (*n* = 1) [171], pulmonary embolism (*n* = 2; with fatal PE in one patient) [167,169], skin necrosis (*n* = 1) [171], arterial stroke (*n* = 1) [168], and aortic thrombosis with peripheral embolization (*n* = 1) [169]. The median platelet count nadir for seven patients was 39 × 10^9^/L (range, 20 to 51; for one patient in whom the platelet count fell from 177 to 75 × 10^9^/L, the nadir platelet count value was not indicated). All seven patients who were tested for anti-PF4 antibodies tested strongly positive in a PF4-dependent ELISA.

The SRA was performed using serum from three of the patients [165,170,171]. All three sera exhibited strong serum-induced serotonin-release at 0 U/mL heparin (HISR > 80%), consistent with the presence of aHIT antibodies. Further, in studies using diluted sera, all three sera showed evidence of increased serotonin-release in the presence of fondaparinux at clinically-relevant concentrations (strongest at 0.1 μg/mL). It has been proposed that the serological picture of HISR, with enhancement in the presence of fondaparinux, could be a serological marker for fondaparinux-induced HIT [170,171]. A confounding feature is that five of the eight cases of fondaparinux-induced HIT cases occurred in patients who were receiving fondaparinux for thromboprophylaxis after knee replacement surgery [165,168,169,171], and knee replacement surgery is a known trigger of SpHIT [for review: 7]. Nevertheless, two cases of convincing fondaparinux-induced HIT occurred in other clinical settings not implicated in SpHIT (post-hip replacement surgery [166]; urosepsis [170]), and for one of these patient sera, the putative serological markers of fondaparinux-induced HIT were also demonstrated [170].

### 3.6. Unusually Severe HIT

Sometimes patients with unusually severe HIT, either with marked thrombocytopenia or with multi-site thrombosis, can be shown to have aHIT antibodies. For example, I previously reported a patient with a typical platelet count nadir of 61 × 10^9^/L who had developed HIT on postoperative day 6 while receiving UFH thromboprophylaxis post-fracture surgery, i.e., a very typical temporal presentation of HIT [60]. However, this patient had numerous thrombotic complications, including multiple arterial thrombi (strokes, limb artery thrombosis) and venous thrombi (pulmonary emboli, bilateral adrenal hemorrhages inferring presence of adrenal vein thromboses), thus presenting an unusually severe multi-site thrombotic presentation of HIT. Serological studies showed marked HISR (~90%), consistent with the diagnosis of aHIT. The clinical course and response to high-dose IVIG is shown later in this article (see Section 3.9.3, High-Dose IVIG).

### 3.7. Laboratory Diagnosis

One of the key tenets of this review is that aHIT antibodies have a serological hallmark, namely their ability to activate platelets in the absence of heparin. This can be best shown by functional assays, such as the SRA [41,79,80], i.e., HISR, or heparin-induced platelet activation (HIPA) test [172,173], where the two laboratories that developed these washed platelet assays (McMaster Platelet Immunology Laboratory and Greifswald University, respectively) routinely perform the assays in the absence of heparin (“buffer control”).

#### 3.7.1. Heparin-Independent Platelet-Activating Properties

In parallel with the growing recognition of aHIT disorders, some research laboratories also provided evidence regarding heparin-independent properties of these antibodies. Importantly, Prechel and coworkers [174] established that this phenomenon was not abrogated by attempts to remove or degrade heparin; in subsequent studies, they showed that surface expression of PF4 improves ability to detect heparin-independent platelet-activating properties [175]. Interestingly, these studies occurred a decade after earlier attempts by Caple and colleagues [176] to abrogate reactivity of HIT sera in the absence of heparin by removing heparin from the blood samples; they found that such heparin removal filters had an unpredictable effect on decreasing reactivity in the absence of heparin. These studies became understandable after the concept of heparin-independent platelet-activating antibodies became established.

Socher and coworkers [177] systematically studied this phenomenon using the HIPA test and found that approximately half of their samples showed some degree of platelet activation in the absence of heparin. In a minority of such samples, residual heparin was the explanation for this phenomenon. Nonetheless, a significant minority (at least 40%) of samples were shown to have heparin-independent platelet-activating properties.

Satoh and colleagues [178] also showed heparin-independent anti-PF4 reactivity in approximately 14% (17/118) of patients with systemic lupus erythematosus; there did not appear to be any correlation between the detection of heparin-dependent and heparin-independent antibodies in these patients. However, none of the patients with heparin-independent antibodies had a positive SRA, so the clinical significance of these observations remains uncertain.

These studies parallel the development of HIT-mimicking monoclonal antibodies. For example, some monoclonal antibodies (e.g., KKO, 5B9) recognize heparin-dependent antigen sites formed on PF4 only in the presence of heparin [179,180]. In contrast, other monoclonal antibodies reactive against PF4 (e.g., 1E12, 1C12, 2E1), designed with a human Fc moiety, resemble more closely human aHIT antibodies, by binding to sites distinct from the heparin-dependent binding sites [181]. Indeed, one of these monoclonal antibodies—2E1—exhibited unique bivalent binding, involving not only the antigen recognition site on PF4 but also charge-dependent interactions with heparin.

Various antigen sites on PF4 have been mapped. At least two antigen sites on PF4 have been identified that are formed in the presence of heparin, and which are recognized by heparin-dependent HIT antibodies and some heparin-dependent monoclonal antibodies; notably, these antigens are distinct from the heparin-binding region on PF4 [182,183,184].

In 2021, Huynh and coworkers identified the VITT antigen binding site as being located on the heparin-binding site of PF4 [185,186]. The formation of PF4-IgG immune complexes can be created without the requirement for heparin. These workers also showed that HIT sera with HISR also recognize antigens at the heparin-binding site [185]; presumably, these binding properties account for the heparin-independent platelet-activating properties caused by aHIT antibodies. Interestingly, these authors also found that risk of CVST differed with respect to whether the VITT antibodies required supplemental PF4 to test positively in the SRA; in essence, sera from patients that did not require supplemental PF4 to cause HISR were more likely to be complicated by CVST [187].

#### 3.7.2. Immunoassays for aHIT Antibodies

It is not possible to distinguish between cHIT and aHIT antibodies using standard HIT immunoassays, such as any of several widely used PF4/polyanion ELISAs, except perhaps indirectly, as aHIT sera tend to have unusually high optical density (OD) values [41,58]. In a fluid-phase ELISA, my research associate, Jo-Ann Sheppard, showed that aHIT sera tended to show stronger reactivity (versus cHIT sera) against PF4 alone, but these differences were not sufficiently great to be diagnostic [15].

A promising new approach is the recent development of a rapid chemiluminescence immunoassay (CLIA) that detects anti-PF4 (not anti-PF4/heparin) antibodies [188]; such an assay could be complementary to the existing, commercially-available, PF4/heparin-CLIA [189,190]. Approximately 30% of HIT sera yielded a positive result in the novel PF4-CLIA—presumably reflecting detection of aHIT antibodies—in addition to the expected positive result in the standard PF4/heparin-CLIA. If validated, and commercialized, the prospect of dual testing with PF4/heparin- and PF4 only CLIAs would optimize diagnostic sensitivity for HIT while at the same time allowing for detection of anti-PF4 reactivities seen in aHIT as well as in VITT and VITT-like disorders.

#### 3.7.3. Technical Challenges

An important technical issue raised by Kanack and colleagues [191] is to what extent false-positive diagnoses of aHIT may be made based on the presence of residual heparin in diagnostic samples. This is relevant because unaccounted for residual heparin could result in inadvertent detection of heparin-dependent antibodies. The authors point out that ongoing heparin administration occurrs in approximately half of patients whose blood is referred for HIT diagnostic testing [192]. Further, one study [58] found residual heparin (at least 0.1 IU/mL) was present in 62% of samples referred for HIT testing. Approaches to minimize the false-positive detection of aHIT antibodies include either treatment of the sample with heparinase or to require a prolonged period (perhaps at least 12 h) from discontinuation of heparin to blood sampling [191]. Despite these reservations, Kanack and colleague did comment that some samples—including those of patients with SpHIT—do exhibit “true aHIT” with heparin-independent platelet activation. Indeed, this same group of investigators has published several examples of aHIT, including a demonstration of heparin-independent properties [56,85].

#### 3.7.4. False-Positive Detection of Cross-Reactivity

Given the prolonged thrombocytopenia frequently seen in aHIT disorders, there could be clinical concern regarding “cross-reactivity” of the HIT antibodies against the anticoagulant being given, especially if this is the sulfated pentasaccharide, fondaparinux, or the low-sulfated glycosaminoglycan, danaparoid. When performing such laboratory studies of in vitro cross-reactivity, using platelet activation assays, it is important to include a “buffer control”, to ensure that any reactivity seen in the presence of drug is indeed greater than control [97]. Our laboratory has used this approach in testing for cross-reactivity, in which comparisons with buffer have shown that in some cases cross-reactivity with fondaparinux is present [170,171], whereas in others it is not [64]. Another approach to assessing cross-reactivity is to perform fluid-phase ELISAs [145].

### 3.8. Pathogenesis of aHIT

**Figure 6:**
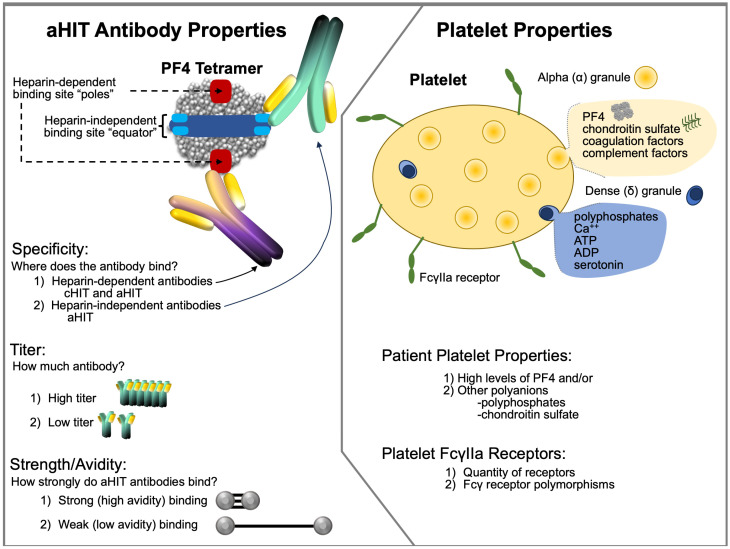
Speculations on aHIT pathogenesis: aHIT antibody properties (left) and platelet properties (right). Abbr.: ADP, adenosine diphosphate; aHIT, autoimmune heparin-induced thrombocytopenia; ATP, adenosine triphosphate; Ca++, calcium ions; cHIT, classic HIT; PF4, platelet factor 4.

It seems most likely that aHIT reflects the formation of highly-pathological HIT antibodies with strong heparin-independent platelet-activating antibodies, i.e., HIT antibodies. However, patient-specific risk factors may also be present. Figure 6 illustrates some of the pathophysiological considerations that could account for aHIT.

#### 3.8.1. aHIT Antibodies

It is clear that aHIT pathogenesis must involve the formation of highly pathological antibodies that are capable of activating platelets independently of the presence of heparin. This conjecture follows logically from a simple observation. As was shown by Table 2, patients who evince atypical HIT suggestive of aHIT generally have platelet activation assay results that show strong platelet activation in the absence of heparin. Most of the reports were from the McMaster Platelet Immunology Laboratory, which utilizes the SRA, and hence HISR is shown. In general, there is strong (>80% serotonin-release) release observed with at least half of these aHIT sera, with most of the remaining sera showing at least a 50% threshold of HISR. Similar observations have been presented by laboratories that utilize another washed platelet activation assay—the HIPA test. Here, platelet aggregation within 5 min is a marker of strong platelet activation, and these workers have also demonstrated such strong activation in the absence of heparin [69,75,77].

A simple classification scheme distinguishes three categories of anti-PF4 antibodies [2,181]. Type 1 antibodies are non-pathogenic despite recognizing PF4/heparin complexes, but are not platelet-activating; these account for false-positive reactions by ELISA. Type 2 antibodies are pathogenic, platelet-activating antibodies that recognize PF4/heparin complexes, i.e., the antibodies are heparin-dependent. Finally, type 3 antibodies refer to highly-pathogenic, heparin-independent antibodies that recognize PF4 without the need for heparin. The current paradigm in the HIT research field is that aHIT sera contain both type 2 and 3 (and potentially also type 1) antibodies, with the heparin-independent type 3 antibodies key to aHIT pathogenesis.

Studies by Greinacher and colleagues have improved the understanding of the binding properties of the type 3 antibodies that underlie aHIT pathogenesis (for review see [193]). These workers used biophysical techniques [194] such as atomic force microscopy (AFM) [148], circular dichroism (CD) spectroscopy [195,196], single-molecule force spectroscopy (SMFS) [197,198], and isothermal titration calorimetry (ITC) [198,199], among others. AFM was used to show that close approximation of the cationic (and thus usually repelling) PF4 tetramers was critical to forming antigens recognized by HIT antibodies [148]. CD spectroscopy provided the first direct evidence for structural changes in PF4 during complex formation with heparin [195]. By combining SMFS and ITC studies, these workers showed that both qualitative and quantitative features of HIT antibodies (both cHIT and aHIT antibodies) are important for HIT antibody pathogenicity [198]. In essence, the greater the binding strength of the antibodies, and the higher their concentration, the greater the platelet-activating potential [198], particularly when considering platelet membrane surfaces rather than artificial surfaces such as ELISA plates [200,201].

These investigators further showed for aHIT antibodies the binding forces are particularly strong (≥100 pN), which are thus able to fuse together two PF4 tetramers even in the absence of heparin molecules [198]. This contrasted with cHIT antibodies, with lower binding forces (60–100 pN), which required heparin for platelet activation. This model also indicated that clustering of PF4 molecules by highly pathogenic type 3 (aHIT) antibodies could also allow for binding of type 2 (heparin-independent) antibodies, even in the absence of heparin, contributing to formation of large PF4-IgG immune complexes capable of strong platelet activation. Although these studies did not address the epitope location on the target protein, PF4, for aHIT antibodies, it is probable that they bind with high affinity to the heparin-binding site on PF4 (see Figure 6).

#### 3.8.2. Patient (Platelet) Risk Factors for aHIT

It also would seem plausible that platelet-dependent factors might also play a role; however, these have not been investigated to date in any detail. For example, PF4 levels differ among platelets [202,203], and—as shown in Table 2—some patients that showed aHIT clinical profiles had relatively weak HISR. It is possible that these patients themselves have substantial platelet-associated PF4 levels, thus magnifying the clinical effect of less marked degrees of aHIT antibodies. Cines and colleagues [204] have proposed that high levels of platelet-associated PF4 could be a risk factor for HIT. Given that the formation of large PF4/heparin multimolecular complexes is crucial to HIT pathogenesis [205,206], it is logical to assume that high PF4 levels could contribute to greater HIT antibody-induced platelet activation irrespective of whether cHIT or aHIT antibodies are involved.

Another possibility is that there are differences among normal individuals in the varieties and quantities of platelet-associated polyanions that may facilitate formation of PF4/aHIT-IgG immune complex formation in the absence of heparin. For example, polyphosphates have been implicated in HIT pathogenesis [196,207], as has chondroitin sulfate [208] (chondroitin sulfate is the predominant glycosaminoglycan present within platelets [209]).

### 3.9. Treatment of aHIT

#### 3.9.1. General Considerations

aHIT has an increased probability of severe clinical outcomes, making treatment more complex. For example, patients may present with CVST (with secondary cerebral hemorrhage), or bilateral adrenal hemorrhage, resulting in special treatment considerations (e.g., relative contraindication to aggressive anticoagulation, need for corticosteroid administration for adrenal insufficiency, and so forth). Some patients develop acute renal failure, potentially complicating anticoagulant therapy. Microvascular thrombosis in the context of overt DIC is also challenging to manage.

The treatment of aHIT differs from the treatment of classic HIT. Heparin cessation (although advised) may not be of benefit, and—counterintuitively—may even be relatively harmful; this is because the heparin-independent platelet-activating antibodies will continue to activate platelets even in the absence of heparin (and any anticoagulant benefit of heparin is lost with its discontinuation). Moreover, the frequency of overt DIC is high in aHIT; this points to relative advantages of Xa inhibition over (APTT-adjusted) DTI treatment for management of aHIT (discussed subsequently). Special treatments—particularly high-dose IVIG, but also sometimes plasma exchange—may be needed. In addition to measuring daily platelet counts, I also advocate for measuring daily fibrinogen and d-dimer levels when treating aHIT, as successful anticoagulation should be associated with stable/rising fibrinogen values and gradually decreasing d-dimer levels.

#### 3.9.2. Choice of Anticoagulation

Many aHIT patients have overt decompensated DIC [41]; some patients develop DIC-related clinical sequelae such as symmetrical peripheral gangrene [91,92]. If aHIT-associated DIC results in relative prolongation of the APTT, it complicates APTT-based monitoring, which is the most common approach to anticoagulation with direct thrombin inhibitor (DTI) therapy. There are numerous examples of DTI failure in the literature (see for example Table 3), some of which are plausible examples of so-called “APTT confounding” [54,76,92,104,210] (Table 2 and Table 3). This term refers to situations where APTT prolongation to supratherapeutic levels results in inappropriate interruption or dose reduction of DTI therapy, due to the elevated APTT levels reflecting not truly elevated drug levels, but rather the combined effects of anticoagulation in patients with APTT prolongation due to DIC and factor consumption [210]. Although one approach is to use more suitable anticoagulation monitoring techniques, these are not widely available.

Another potential drawback of DTI therapy in aHIT patients with overt DIC is the inhibition of protein C activation; just as warfarin was shown to promote microthrombosis (venous limb gangrene) in some patients with HIT through depletion of the natural anticoagulant, protein C [211], it is also plausible that DTI therapy could (counterintuitively) contribute to microthrombosis in some patients with severe aHIT-associated DIC through the inhibition of (endothelial thrombomodulin-catalyzed) thrombin-induced activation of protein C [212,213].

The author advocates for factor Xa-inhibiting therapies, such as fondaparinux, danaparoid, or a Xa-inhibiting direct oral anticoagulant (DOAC), e.g., rivaroxaban, apixaban [214]. However, sometimes aHIT patients exhibit in vitro and in vivo cross-reactivity with fondaparinux (discussed previously in Section 3.5. Fondaparinux-Associated HIT), although this appears to be uncommon.

#### 3.9.3. High-Dose IVIG

For patients with aHIT, treatment with high-dose IVIG can be helpful, by inhibiting aHIT antibody-induced platelet activation and associated hypercoagulability [61,215,216,217,218]. Dosing should be based on actual (rather than ideal) weight, i.e., 1 g per kilogram on two consecutive days, with an option for a third partial or full dose, if response is suboptimal (although some patients benefit from lower doses or require higher doses for effect). Not all patients respond to IVIG, however, and so therapeutic plasma exchange (TPE) is an option for IVIG-refractory patients (discussed subsequently). IVIG is also indicated for patients with other anti-PF4 disorders that feature heparin-independent platelet-activating antibodies, such as VITT [219,220,221] and SpHIT [222,223].

**Figure 7:**
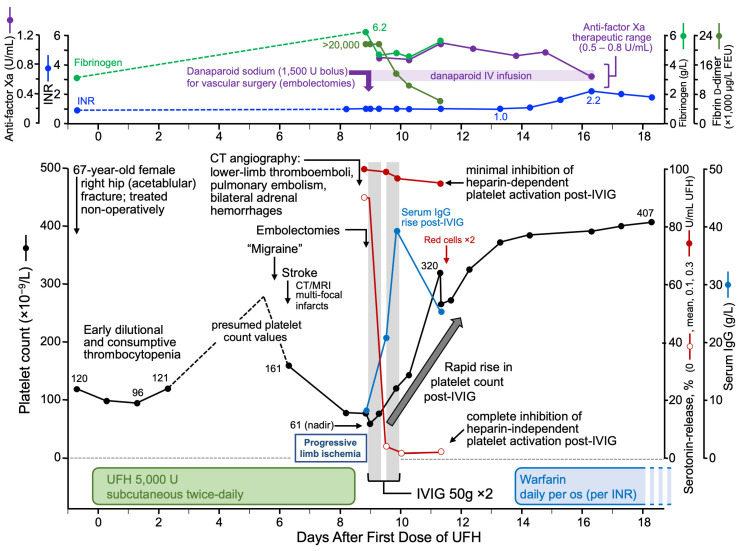
Excellent patient response to high-dose IVIG with parallel decrease in HISR. From [61], with permission (copyright 2019 Taylor and Francis, Milton Park, Oxfordshire, UK). Abbreviations: CT, computed tomography; INR, international normalized ratio; IV, intravenous; IVIG, intravenous immunoglobulin; MRI, magnetic resonance imaging; U, unit; UFH, unfractionated heparin.

Figure 7 shows an example of a patient who was successfully treated with IVIG [61]; the platelet count rose rapidly after IVIG administration; laboratory investigations showed HISR by patient serum that was promptly inhibited following IVIG.

#### 3.9.4. Therapeutic Plasma Exchange (TPE)

TPE is a modality sometimes used to manage severe aHIT, although its most frequent application is to reduce HIT antibody levels prior to planned heparin exposure for cardiac surgery in a patient with recent or acute HIT [217]. In the author’s opinion, the replacement fluid should be frozen plasma (rather than albumin), for at least two reasons. First, patients with severe HIT-associated DIC can have coagulation factor depletion—including natural anticoagulants (protein C, antithrombin)—which could contribute to microvascular ischemia, and frozen plasma administration could ameliorate these abnormalities. Second, FP (but not albumin) contains IgG, and maintaining adequate IgG levels may assist in inhibiting FcγIIa-mediated platelet activation [224].

#### 3.9.5. Heparin Rechallenge and Monitoring for aHIT

Heparin (UFH) rechallenge is the recommended approach for managing cardiac or vascular surgery (and probably also hemodialysis) despite a previous history of HIT (for review see [225]). In theory, for a patient with a history of HIT who no longer has HIT antibodies at time of heparin re-exposure (and who receives no additional postoperative heparin following planned re-exposure for cardiac or vascular surgery), the only way that recurrent HIT can occur is if aHIT develops [66,225]. Accordingly, platelet count monitoring for at least the first 10 days should be performed, looking for unexpected platelet count declines during the day 5 to 10 day “window” characteristic of HIT.

## 4. Discussion

**Figure 8:**
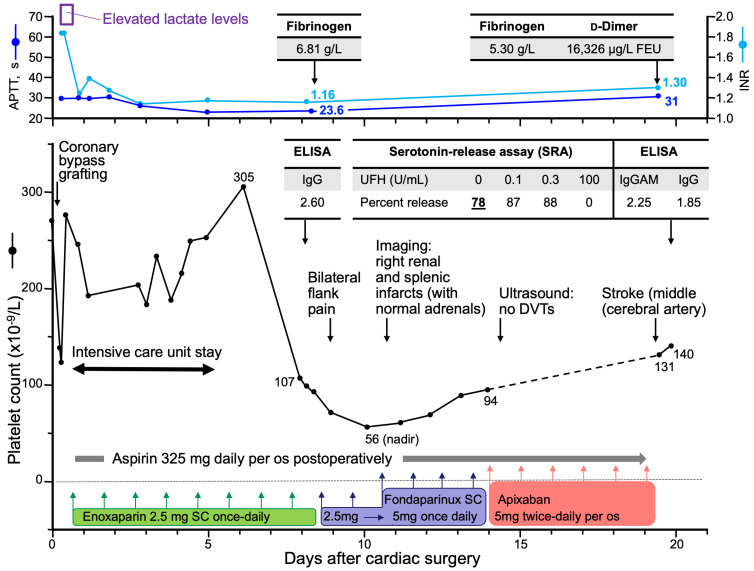
Clinical scenario of autoimmune heparin-induced thrombocytopenia (aHIT). aHIT was diagnosed based upon prolonged platelet count recovery and heparin-independent serotonin-release (78% serotonin-release at 0 U/mL heparin). Abbreviations: APTT, activated partial thromboplastin time; sc, subcutaneous; DVT, deep venous thrombosis; ELISA, (PF4/polyanion) enzyme-linked immunosorbent assay; INR, international normalized ratio; U, unit; UFH, unfractionated heparin.

aHIT presents major diagnostic and treatment challenges. The atypical clinical picture can make diagnosis difficult, and the paucity of laboratories offering tests to document aHIT reactivity contributes to underappreciation of this clinical entity. Moreover, standard treatment approaches for cHIT are often unsuccessful for aHIT. Figure 8 illustrates the challenges of managing aHIT. In this scenario, the patient undergoes prompt treatment for postoperative HIT, and is discharged home on therapeutic-dose DOAC (apixaban); nonetheless, a fatal stroke occurs on postoperative day 20. With the benefit of hindsight, the slow platelet count recovery was an indicator of aHIT, a feature that was confirmed by the SRA, showing substantial (78%) HISR. The presence of documented renal and splenic infarcts approximately 10 days earlier suggests that intra-cardiac thrombosis, with embolization, was likely present. Regardless of the precise explanation for the subsequent stroke, the potential for morbidity and mortality resulting from this severe subtype of HIT is evident.

To conclude this review of aHIT, I list several bulleted comments:

- Autoimmune HIT (aHIT) presents with an atypical clinical picture (e.g., onset or worsening of thrombocytopenia despite stopping heparin; slow platelet count recovery after stopping heparin; multi-site thromboses; unusual sites of thrombosis [CVST, mesenteric vein, adrenal vein/adrenal necrosis/hemorrhage]; microthrombosis [e.g., venous limb gangrene, symmetrical peripheral gangrene]; overt DIC, and so forth).
- aHIT features highly pathological antibodies with heparin-independent platelet-activating properties (type 3 anti-PF4 antibodies).
- aHIT antibodies appear to recognize the heparin-binding site of PF4 (i.e., aHIT antibodies resemble VITT antibodies in this respect).
- HIT laboratories should be encouraged to perform tests that can demonstrate heparin-independent platelet-activating properties, e.g., performing the SRA (or another platelet activation assay) in the absence of heparin (0 U/mL heparin, or “buffer control”); the addition of PF4 may be required in some instances to optimize detection of the heparin-independent antibodies.
- Laboratories with a clinical and/or research interest in HIT should collaborate to determine whether standardization of the SRA or other platelet activation assays can be achieved, so as to help make the diagnosis of HIT more consistent.
- Anticoagulation: factor Xa inhibitors may have advantages over direct thrombin inhibitors (e.g., avoiding risk of APTT confounding).
- In addition to the frequent measurement of platelet counts, regular d-dimer and fibrinogen levels should also be assessed when managing a patient with aHIT, as a way to gauge whether HIT hypercoagulability is being adequately controlled (fibrinogen levels should be stable or rising, and d-dimer levels should be steadily decreasing, if a patient with aHIT is well-anticoagulated with an alternative non-heparin anticoagulant).
- Adjunct therapies: high-dose IVIG is an important option to de-escalate hypercoagulability in aHIT disorders, with a rapid platelet count increase (if observed) an indirect marker of IVIG efficacy.
- Therapeutic plasma exchange (TPE) is a potential treatment option for IVIG-refractory patients.

**Figure 9:**
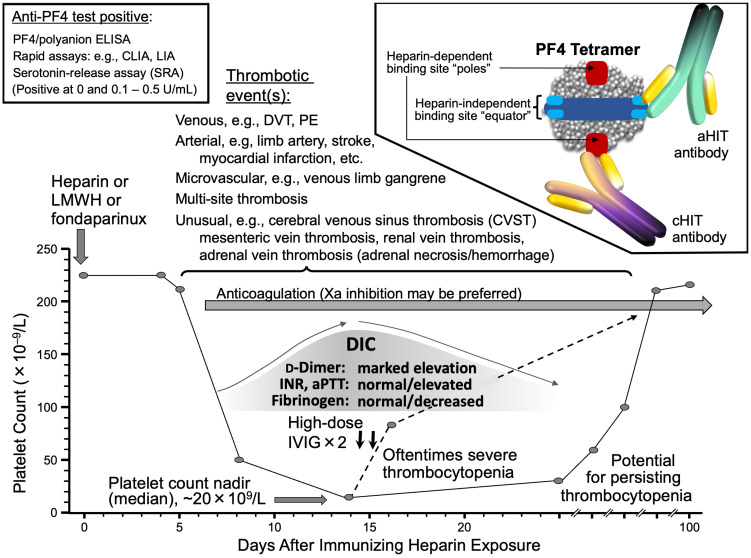
Autoimmune HIT (aHIT): clinical picture and pathogenic aHIT antibodies. The Figure illustrates several key concepts in aHIT, including: oftentimes severe thrombocytopenia, a high frequency of concomitant disseminated intravascular coagulation (DIC), high frequency (probably >90%) of associated thrombotic events (including multiple and/or atypical thromboses), and potential for persisting thrombocytopenia despite cessation of heparin. Patients typically test anti-PF4/polyanion immunoassay-positive (ELISA, CLIA, LIA); in functional (platelet activation) assays, e.g., serotonin-release assay, patients show evidence for both heparin-dependent and heparin-independent platelet-activating antibodies. Two types of HIT antibodies can be detected in aHIT patients: classic heparin-dependent (cHIT) antibodies (cHIT), as well as atypical heparin-independent aHIT antibodies. The author’s preference is to treat aHIT with Xa-inhibitory anticoagulant therapies, along with high-dose intravenous immune globulin (IVIG). Abbr.: aHIT, autoimmune heparin-induced thrombocytopenia; aPTT, activated partial thromboplastin time; cHIT, classic heparin-induced thrombocytopenia; CLIA, chemiluminescence immunoassay; DVT, deep venous thrombosis; ELISA, enzyme-linked immunosorbent assay; INR, international normalized ratio; IVIG, intravenous immune globulin; LIA, latex-enhanced immunoturbidimetric assay; PE, pulmonary embolism; PF4, platelet factor 4; U, units.

I conclude with Figure 9, which summarizes many of the key points made in this review article dealing with aHIT.

## Figures and Tables

**Table 1:** Five autoimmune HIT (aHIT) disorders.

Name of aHIT Disorder	Concept (Definition)
Delayed-onset HIT	Platelet count fall that begins or worsens despite stopping heparin
Persisting (refractory) HIT	Delayed platelet count recovery despite stopping heparin (>1 week)
Heparin “flush” HIT	HIT that occurs with exposure only to small amounts of heparin
Fondaparinux-induced HIT	HIT associated with proximate exposure to fondaparinux
Unusually severe HIT	Marked thrombocytopenia, multiple-site thromboses, overt DIC, etc.

Abbreviations: aHIT, autoimmune heparin-induced thrombocytopenia; DIC, disseminated intravascular coagulation; HIT, heparin-induced thrombocytopenia.

**Table 2:** Studies showing the role for aHIT antibodies (e.g., heparin-independent serotonin-release [HISR] in aHIT).

Study	Key Observations, Including Heparin-Independent Serotonin-Release (HISR)	HISR (%)
	*Case-series with controls*	
[41]	Greater HISR in 12 aHIT patients vs. 24 HIT controls (*p* < 0.05)	50% (mean)
[54]	Delayed platelet count recovery: aHIT vs. cHIT (5/5 vs. 1/6; *p* = 0.015)	>50%
[55]	Higher frequency of HISR in aHIT pts vs. controls (4/4 vs. 34/100; *p* = 0.016)	>80%
[56]	3 cases with SRA-positive refractory HIT (IVIG-responsive)	>40% PEA ^[low PF4] a^
[57]	3/3 pts with post-discharge HIT had HISR (80%, 83%, 99%)	>80%
[58]	aHIT: higher thrombosis rate, lower platelet nadirs, slower platelet count recovery	≥30%
	*Relationship between platelet counts and HISR (serial blood samples)*	
[59]	Inverse relationship between HISR and platelet count (*n* = 1 pt)	>80% (peak)
[55]	Inverse relationship between HISR and platelet count (*n* = 2 pts)	>90% (peak)
[60]	Inverse relationship between HISR and platelet count (*n* = 1 pt)	>90% (peak)
[61]	Abrupt plt count rise and parallel decrease in HISR post-IVIG (*n* = 1 pt)	90% (peak)
	*aHIT pts (case reports) with laboratory evidence of HISR*	
[52]	Delayed/persisting with DIC, microvascular ischemia, nadir = 2	>80% ^b^
[62]	Delayed; warfarin-induced microthrombosis (see Figure 3A), nadir = 71	>40%
[63]	Delayed; postoperative platelet count fall, DVT (see Figure 3B), nadir = 28	>40%
[64]	Delayed/persisting; stroke, adrenal hemorrhages, DVT, DIC, nadir = 68	>90%
[65]	Delayed, DVT, while on Fx; serial rise and fall of HISR, nadir = 13 (post-UFH)	>70%
[66]	Delayed/persisting, recurrent (i.e., repeat UFH; previous HIT), DVT, nadir = 20	>90%
[67]	Delayed, adrenal hemorrhages, DVT, nadir = 117	>90%
[68]	Delayed/severe, DIC, multilimb microvascular gangrene, death; nadir = 10	>90%
[69]	Delayed, persisting, DVTs, Fx cross-reactivity, nadir = 3	HIPA + 5 min ^c^
[70]	Delayed/persisting; no thrombosis (ascribed to rivaroxaban), nadir = 56	>70%
[71]	Severe, DIC with coagulation factor depletion, death, nadir = 65	>60%
[72]	Delayed/persisting, DIC, microvascular limb ischemia, death, nadir = 16	>80%
[73]	Delayed/persisting, DVTs (argatroban failure), nadir = 16	100%
[74]	Delayed, multiple arterial/venous (incl. adrenal) thrombi, IVIG, nadir = 10	100%
[75]	Delayed, CVST, DIC, argatroban failure, death, nadir = 7	HIPA + ^c^
[76]	Delayed/persisting, venous limb gangrene (bivalirudin failure), IVIG, nadir = 9	100%
[77]	Delayed/persisting, DVT, IVIG, nadir = 12	HIPA + 5 min ^c^
[78]	Persisting, multiple venous/arterial thrombi (argatroban failure), IVIG, nadir = 25	ATP release ^c^

Footnotes: ^a^ Positive PEA result occurred at low PF4 concentrations; ^b^ Primary report did not include data on HISR. ^c^ Platelet activation test was strongly positive in the absence of heparin. Abbreviations: aHIT, autoimmune HIT; ATP, adenosine triphosphate; cHIT, classic HIT; CVST, cerebral venous sinus thrombosis; DIC, disseminated intravascular coagulation; DVT, deep venous thrombosis; Fx, fondaparinux; HIPA, heparin-induced platelet activation test; IVIG, high-dose intravenous immunoglobulin; PEA, platelet factor 4 (PF4)-dependent platelet activation assay; plt = platelet count; pt, patient; UFH, unfractionated heparin; +, positive.

**Table 3:** Reported cases of aHIT without laboratory demonstration of aHIT antibodies (“Refractory”, >7-day plt recovery).

Study	Trigger	Nadir	HIT Thrombosi(e)s	Treatment	Other	Refractory
[45]	U-cpb	51 ^a^	LAT	Warf, aspirin	Post-D/C; amp	
[45]	U-cpb	29	DVT	DS	Post-D/C	
[45]	U-cpb	25	LAT	DS, Lep	Post-D/C; amp	Yes
[45]	U-cpb	40	DVT, PE	Lep; IVC filter	Post-D/C	
[82]	U-rx	15 ^a^	DVTs × 3	Lep; IVC filter	Post-D/C	
[82]	U-cpb	54 ^a^	SVG-thrombosis × 1	Lep	Post-D/C	
[83]	U-cpb	7	VLG × 2 limbs	Arg	VLG (Warf)	Yes
[83]	U-cpb	18	VLG × 2 limbs	Lep	VLG (Warf)	Yes
[84]	U-vasc	5	DVT; graft thrombus	Arg, CS, IVIG	Arg-fail (DVT)	Yes
[84]	U-vasc	16	DVTs × 2	Arg, CS, IVIG, Fx	DIC	Yes
[85]	U-rx	19	DVT	Arg, Fx, CS, IVIG	In Vitro IVIG studies	Yes
[85]	U-pr	18	DVT	Arg, IVIG	In Vitro IVIG studies	Yes
[86]	U-rx	7	DVT, PE, ecchymoses	IVC filter, Warf, IVIG	DIC	Yes
[87]	U-rx	24	DVT, purpura	Warf	DIC	Yes
[88]	U-pr	7	CVST, DVT, PE	Nil	Post-D/C, DIC	Yes
[89]	U-cpb	39	DVT × 2, VLG × 2	Lep	Post-D/C; amps	
[90]	U-cpb	69	MI, SVG-thrombosis × 4	Lep	Post-D/C	
[91]	U-cpb	8	DVT, SPG × 3 limbs	Arg	Amps × 3 limbs	Yes
[92]	U-cpb	40	PE; SPG × 4 limbs	Lep	DIC, Lep-fail; amps	Yes
[93]	L-pr	58 ^a^	PE	Arg	Post-D/C; death	Yes
[94]	U-cpb	3	Ecchymoses	Arg	No sequelae	Yes
[95]	U-rx	8	DVT progr, IVC filter	Arg, Lep, CS, TPE, Ritux	Lep/Arg-fail, amps × 2	Yes
[96]	L-pr	23	DVT, PE	Fx, DS, Riv	Fx/DS-fail (↑dD)	Yes
[97]	U-cpb	12	Testicular, ^b^ PE	DS, Biv, TPE	X-R assays conducted	Yes
[98]	U-cpb	8	LAT, DVT/PE, Lt atrial	Arg, Biv, CS, IVIG	Post-D/C	Yes
[99]	U-rx	26	Nil HIT thrombosis	IVC filter, Fx, Arg, IVIG	No sequelae	Yes
[100]	U-hd	15	DVT	Arg, IVIG, apix	Post-D/C	Yes
[101]	L-pr	25	CVST	Arg, CS, IVIG, Warf	Post-D/C	Yes
[102]	L-pr	<10	CVST, DVT	Arg, Biv, CS, IVIG	Arg-fail (worse DVT)	Yes
[103]	U-hd	16	DVT	Arg, IVIG, Apix	Arg-fail (DVT)	Yes
[104]	U-pr	4	DVTs × 4 limbs	Arg, Biv, CS, IVIG	Arg-fail (DVT ischemia)	Yes
[105]	L-pr	32 ^a^	CVST	Biv	Post-D/C	
[106]	U-pr	36	LAT, PE, PFO ^c^	Arg, IVIG	Post-D/C	
[107]	U-MI	~100	DVT, LAT	Heparin continued	Amp × 1	
[108]	U-pr	28	DVT	Arg, tPA, IVIG	Post-D/C	Yes
[109]	U-pr	<12	DVT × 3, PE	Riv	Post-D/C	
[110]	L-pr	8	Bilateral LAT	Arg, IVIG	Post-D/C	
[111]	U-cpb	24	Strokes (arterial)	Arg, IVIG	DIC; Arg fail (↑dD)	Yes

^a^ Platelet count nadir occurred after resumption of heparin. ^b^ Testicular vein thrombosis. ^c^ PFO (patent foramen ovale) device thrombosis. Abbreviations: Amp, amputation; Apix, apixaban; Arg, argatroban; Biv, bivalirudin; CS, corticosteroids; CVST, cerebral venous sinus thrombosis; ↑dD, elevated d-Dimer levels; DIC, disseminated intravascular coagulation; DS, danaparoid, sodium; DVT, deep-vein thrombosis; fail, failure; Fx, fondaparinux; IVC, inferior vena cava; IVIG, high-dose intravenous immunoglobulin; LAT, limb artery thrombosis; Lep, lepirudin; L-pr, prophylactic-dose low-molecular-weight heparin; MI, myocardial infarction; PE, pulmonary embolism; Post-D/C: HIT presenting after discharge from hospital; Riv, rivaroxaban; SPG, symmetrical peripheral gangrene; SVG, saphenous vein graft; tPA, tissue-plasminogen activator; TPE, therapeutic plasma exchange; U-cpb, UFH given during cardiopulmonary bypass (heart) surgery; U-hd, heparin for hemodialysis; U-MI, heparin for myocardial infarction; U-Pr, prophylactic-dose heparin; U-rx, therapeutic-dose (treatment-dose) heparin; vasc, vasular surgery; VLG, venous limb gangrene; Warf, warfarin; X-R, cross-reactivity.

## Data Availability

Not applicable.
